# Rare connective tissue diseases in patients with C1-inhibitor deficiency hereditary angioedema: first evidence on prevalence and distribution from a large Italian cohort study

**DOI:** 10.3389/fimmu.2024.1461407

**Published:** 2024-10-18

**Authors:** P. Triggianese, R. Senter, F. Perego, A. Gidaro, A. Petraroli, F. Arcoleo, L. Brussino, F. Giardino, O. Rossi, D. Bignardi, P. Quattrocchi, R. Brancaccio, A. Cesoni Marcelli, P. A. Accardo, L. Lo Sardo, E. Cataudella, M. D. Guarino, D. Firinu, A. Bergamini, G. Spadaro, A. Zanichelli, M. Cancian

**Affiliations:** ^1^ University of Rome Tor Vergata, “Fondazione PTV Policlinico Tor Vergata”, Rome, Italy; ^2^ Department of Medicine, Azienda Ospedale-Università di Padova, Padova, Italy; ^3^ IRCCS Istituti Clinici Scientifici Maugeri, Milano, Italy; ^4^ Internal Medicine, Department of Biomedical and Clinical Sciences, Luigi Sacco Hospital, ASST Fatebenefratelli-Sacco, University of Milan, Milan, Italy; ^5^ Department of Internal Medicine, Clinical Immunology, Clinical Pathology and Infectious Disease, Azienda Ospedaliera Universitaria Federico II, Napoli, Italy; ^6^ Ospedali Riuniti Villa Sofia-Cervello, Unità Operativa Complessa di Patologia Clinica, Palermo, Italy; ^7^ Allergy and Clinical Immunology Unit, Department of Medical Sciences, University of Torino & Mauriziano Hospital, Torino, Italy; ^8^ Azienda Ospedaliero-Universitaria Policlinico “G.Rodolico-San Marco”, Catania, Italy; ^9^ Immunoallergology Unit, University Hospital of Careggi, Florence, Italy; ^10^ Department of Medicine Integrated with the Territory, Ospedale Policlinico San Martino, IRCCS Ospedale Policlinico, Genova UO Allergologia, Genova, Italy; ^11^ Department of Clinical and Experimental Medicine, School and Operative Unit of Allergy and Clinical Immunology, University of Messina, Messina, Italy; ^12^ Dermatology Unit, Azienda Unità Sanitaria Locale-IRCCS di Reggio Emilia, Reggio nell’Emilia, Italy; ^13^ Allergy Unit, Civitanova Marche, Italy; ^14^ Division of Allergy and Clinical Immunology, University of Cagliari, Cagliari, Italy; ^15^ Operative Unit of Medicine, Angioedema Center, IRCCS Policlinico San Donato, San Donato Milanese, Milan, Italy; ^16^ Department of Biomedical Sciences for Health, University of Milan, Milan, Italy

**Keywords:** antiphospholipid, autoimmunity, complement, connective tissue diseases, hereditary angioedema, scleroderma, Sjogren syndrome, systemic lupus erythematosus

## Abstract

**Introduction:**

In patients with Hereditary Angioedema (HAE) related to primary C1 inhibitor deficiency (C1INH), the defective clearance of immune complexes and apoptotic materials along with impairment of normal humoral response potentially leads to autoimmunity. Few studies report evidence on autoimmune diseases in C1INH-HAE, but no large population studies focus on rare connective tissue diseases (RCTDs). We aim at evaluating for the first time prevalence and distribution of RCTDs - Systemic Lupus Erytematosus (SLE), primary Sjogren Syndrome (SjS), primary antiphospholipid syndrome (APS), Systemic Sclerosis (SSc), and mixed connective tissue diseases (MCTD) in a large Italian cohort of C1INH-HAE patients.

**Methods:**

A multicenter observational study includes C1INH-HAE patients from ITACA Centers throughout Italy (time frame Sept 2023-March 2024). Inclusion criteria are i. a defined diagnosis of type I or type II C1INH-HAE; ii. age ≥15 years (puberty already occurred); iii. enrollment in the ITACA Registry. The diagnosis of SLE, primary SjS, primary APS, SSc, and MCTD are made in accordance with international classification criteria.

**Results:**

Data are collected from a total of 855 C1INH-HAE patients referring to 15 ITACA Centers. Patients with concomitant RCTDs were 18/855 (2.1%) with F:M ratio 3.5 and a prevalent type I C1INH-HAE diagnosis (87.2%). A diagnosis of SLE results in 44.5% of cases (n=8) while the remaining diagnoses are primary SjS (22.2%, n=4), primary APS (16.6%, n=3), SSc (11.2%, n=2), and a single case of MCTD (5.5%). The female gender is prevalent in all the RCTDs. Patients on long term prophylaxis (LTP) are significantly prevalent in RCTDs group than in the whole C1INH-HAE population (p<0.01).

**Conclusions:**

A relevant prevalence of RCTDs is documented in C1INH-HAE patients, mainly SLE. Patients with RCTDs are on LTP in a significant proportion supporting the idea of a bidirectional link between C1INH-HAE and autoimmunity.

## Introduction

Hereditary angioedema (HAE) resulting from the genetic defect of C1 inhibitor (C1INH) is a rare autosomal dominant disease characterized by recurrent attacks of submucosal and cutaneous edema: angioedema attacks can potentially involve any site and generally are self-limiting within 72 hours (1, 2). The defect of C1INH can be either a quantitative deficiency (type I C1INH-HAE) or a qualitative dysfunction (type II C1INH-HAE) of the protein: the overproduction of bradykinin (BK) related to the lack of activity of C1INH leads to a consequent abnormal activation of the BK-B2 receptors (3) resulting in an increased vascular permeability and, thus, angioedema attacks (2). In disorders due to deficiencies of complement system (CS) components and related regulatory proteins, immune complexes (IC) mediated diseases could be promoted because of the defect of early components like C1q, C1r, C1s, C4, and C2 (4, 5). That network could lead to lupus-like syndromes as occur in patients with primary genetic deficiencies of C2, C4, and other complement components (6, 7). Along with the constitutively reduced levels of C4 and C1INH, sex hormones, mainly estrogens, pathogenetically link autoimmune diseases and C1INH-HAE (8–11). As known, systemic autoimmune diseases have a clear female preponderance and can mainly arise following pubertal onset strengthening the key role for sex hormones in autoimmunity (10, 11). In addition, C1INH-HAE women experience a stronger disease severity with poorer quality of life than males (12–16) as occur in patients affected by systemic autoimmune diseases including Systemic Lupus Erythematosus (SLE) and antiphospholipid syndrome (APS) (17–21). Occasional reports describe potential links between autoimmune conditions and C1INH-HAE (22–25). Although several immunoregulatory disorders have been documented, the specific prevalence of autoimmune diseases in patients with HAE remains debated (9, 26, 27). To date, no studies have specifically focused on the occurrence of rare connective tissue diseases (RCTDs) in C1INH-HAE (23).

Therefore, we aim at evaluating distribution of RCTDs including SLE, primary Sjögren’s syndrome (SjS), primary APS, Systemic Sclerosis (SSc), and mixed connective tissue disease (MCTD) in C1INH-HAE patients providing first evidence from a large Italian cohort study.

## Patients and methods

A multicenter observational study includes C1INH-HAE patients from ITACA Centers throughout Italy (time frame Sept 2023-March 2024). Inclusion criteria are i. a defined diagnosis of type I or type II C1INH-HAE (2); ii. age ≥15 years (puberty already occurred); iii. enrollment in the ITACA Registry, approved by the ethics committee of the coordinating center (Comitato etico Milano area 1) on 5 May 2017 (28).

Data from patients comprise: HAE disease history (age at onset, familiar history, disease activity, treatments’ regimens); demographic and biochemical data (including autoantibodies); the occurrence of a defined diagnosis of RCTDs performed from expert Immunologists/Rheumatologists according to international classification criteria (23). RCTDs include: SLE (29), primary SjS (30), primary APS (31), SSc (32), and MCTD (33). All patients provide written consent to be included in the registry; for patients younger than 18 years, consents are obtained in the presence and with the consent of parents.

Data are collected from a dedicated electronic database for the analyses: patients with relevant clinical missing data (over 20% data missing) are excluded from the study.

### Statistical analysis

Mean and standard deviation (SD) express normally distributed variables; for non-symmetric distributed data, median and range are used. Categorical variables are compared using the Chi-squared test or Fisher’s exact test. P values < 0.05 are considered significant. All statistical analyses are performed using GraphPad Prism version 9 (GraphPad software).

## Results

Data are collected from 15 ITACA Centers: 92.5% of patients (n=855) are included in the study while 7.5% are excluded for clinical missing data. No significant gender prevalence results in the whole cohort with F:M ratio 1.5 (**Table 1**). Type I C1INH-HAE diagnosis is prevalent (87.2%); most of patients have positive familial history while *de novo* mutations are registered in 14% of cases (n=120). A third of cohort at the enrollment results on LTP regimen (**Table 1**).

**Table 1:** Patients from the study population.

	Study Population N=855	Patients with RCTDs N=18
Female (N/%)	518/60.6	14/77.8
F:M ratio	1.5	3.5
Age at the enrollment (yrs) mean ± SD	50.2 ± 25.5	49.7 ± 14.2
Type I HAE (N/%)	745/87.2	16/88.9
Type II HAE (N/%)	110/12.8	2/11.1
Familial history (N/%)	735/85.9	16/88.9
Caucasic (N/%)	848/99.2	17/94.5
ODT only at the enrollment (N/%)	555/65	6/33.3
LTP at the enrollment (N/%)	300/35	12/66.7

RCTDs, rare connective tissue diseases; ODT, on demand therapy; LTP, long term prophylaxis.

### C1INH-HAE patients with concomitant RCTDs

**Figure 1:**
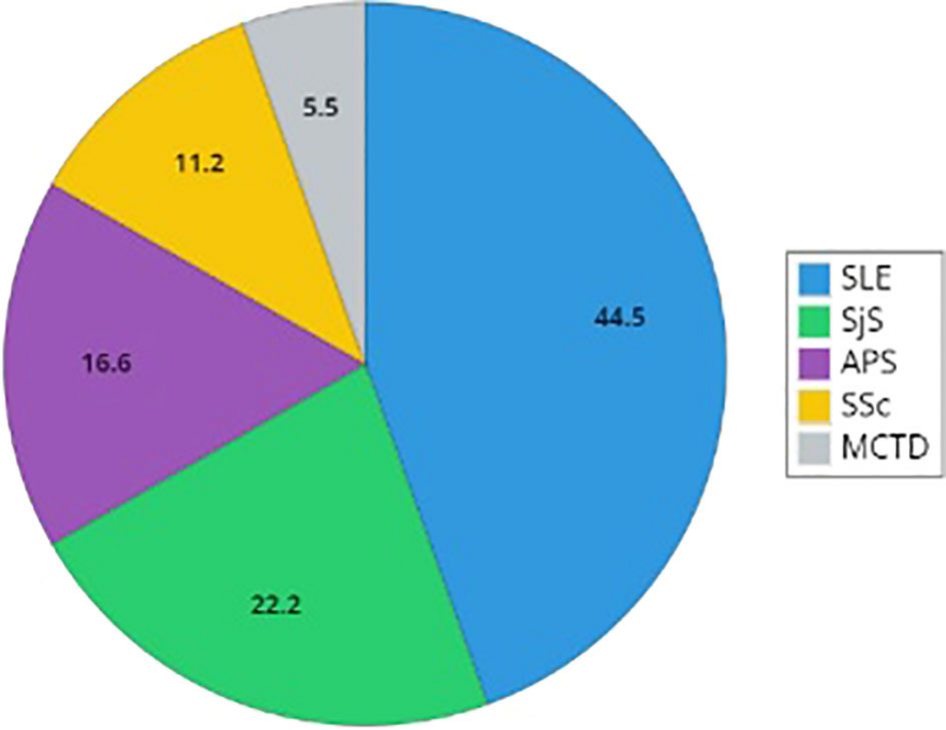
Distribution of rare connective tissue diseases in patients with C1INH-Hereditary Angioedema. The graph reports the distribution of diagnosis of Systemic Lupus Erythematosus (SLE), Sjogren Syndrome (SjS), primary antiphospholipid syndrome (APS), Systemic Sclerosis (SSc), and mixed connective tissue disease (MCTD). Percentages describe the proportion of the population with a defined diagnosis (SLE, primary SjS, primary APS, SSc, and MCTD).

**Figure 2:**
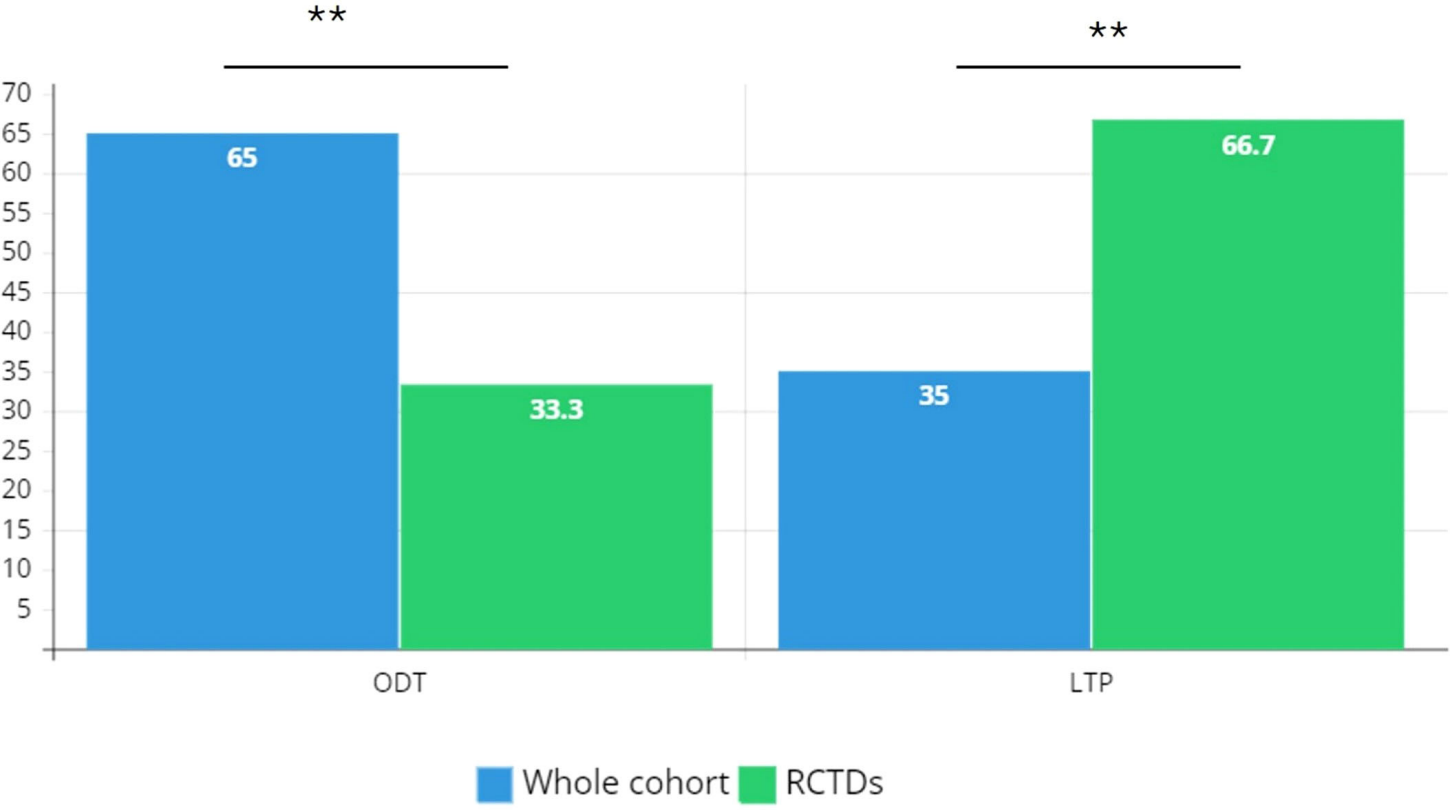
Distribution of treatment regimens in the study cohort. RCTD, rare connective tissue diseases; ODT, on demand therapy; LTP, long term prophylaxis. Chi-square test is used for association between categorical variables; ** P ≤ 0.01.

A diagnosis of RCTDs occurs in 2.1% of the study cohort (n=18): patients with RCTDs are primarily women (n=14) with F:M ratio 3.5 (**Table 1**). In RCTDs patients, type I C1INH-HAE diagnosis is prevalent (83.3%) as well as the positive familial history while *de novo* mutations occur in 11% of cases (n=2). Distribution of diagnoses are reported in **Figure 1**. Patients on LTP are significantly prevalent in RCTDs group than in the whole study population (**Figure 2**). In RCTDs-patients, LTP is already administered at the time of RCTDs diagnosis in 58.4% of cases (7/12): in these cases, attenuated androgens represent the 85.7% (mean dosage 61.2 ± 18 g/year) while plasma derived C1INH-LTP regimen occurs in a single case.

### Prevalence and distribution of RCTDs

In most cases, RCTD diagnoses are made after the diagnosis of C1INH-HAE (**Table 2**). SLE is the prevalent diagnosis (44.5%, n=8), while the remaining diagnoses are primary SjS (22.2%, n=4), primary APS (16.6%, n=3), SSc (11.2%, n=2), and a single case of MCTD (5.5%) (**Figure 1**).

**Table 2:** Case series of patients with hereditary angioedema and concomitant rare connective tissue diseases.

Patient	Sex	Age (yrs)	Ethnicity	Type HAE	HAE Familial history (Y/N)	RCTD	Systemic Involvement	HAE treatments at the time of RCTD diagnosis
1	F	40	Caucasic	I	Y	MCTD	skin, lung, PAH	LTP
2	F	58	Caucasic	I	Y	SSc	diffuse cutaneous SSc	LTP
3	M	65	Caucasic	II	Y	SSc	limited cutaneous SSc	LTP
4	M	44	Caucasic	I	Y	APS	RTE	LTP
5	F	65	Caucasic	I	Y	APS	RTE	LTP
6	F	49	Caucasic	I	Y	APS	Pregnancy morbidity	ODT only
7	F	54	Caucasic	I	N	SjS	SS, PNS	ODT only
8	F	34	Non Caucasic	II	N	SjS	SS, joint	ODT only
9	F	74	Caucasic	I	Y	SjS	SS	LTP
10	F	61	Caucasic	I	Y	SjS	SS	LTP
11	M	40	Caucasic	I	Y	SLE	skin, BC	ODT only
12	F	39	Caucasic	I	Y	SLE	skin, BC	ODT only
13	F	30	Caucasic	I	Y	SLE	skin, joint	ODT only
14	F	39	Caucasic	I	Y	SLE	skin	LTP
15	F	48	Caucasic	I	Y	SLE	kidney, skin, joint	LTP
16	F	26	Caucasic	II	Y	SLE	kidney, skin	LTP
17	M	56	Caucasic	I	Y	SLE	kidney, joint, BC	LTP
18	F	67	Caucasic	I	Y	SLE	kidney, skin, joint, BC	LTP

HAE, hereditary angioedema; RCTD, Rare Connective Tissue Disease; LTP, long term prophylaxis; ODT, on demand therapy; MCTD, Mixed Connective Tissue Disease; PAH, pulmonary arterial hypertension; SSc, Systemic Sclerosis; APS, Antiphospholipid Syndrome; SLE, Systemic Lupus Erythematosus; SS, Sicca Syndrome; RTE, recurrent thrombotic events; PNS, peripheral nervous system; BC, blood cells.

In SLE patients, female gender is common with F:M ratio 3; the mean age at SLE diagnosis is 21 ± 8.3 years, and in 2 patients (1 man and 1 woman) SLE diagnosis is performed 6.5 ± 2 years before C1INH-HAE diagnosis. SLE patients are on LTP in 62.5% of cases (n=5): 2 patients (40%) have another concomitant autoimmune disease, including atrophic gastritis and autoimmune thyroiditis. The main systemic manifestations among SLE patients are skin signs (87.5%) and, in a similar percentage, mild/moderate renal abnormalities and SLE-related cytopenias (50% for both), treated with immunosuppressors (steroids and disease-modifying antirheumatic drugs - DMARDs) in accordance with international recommendations for SLE treatment. All SLE patients are in a stable disease remission (≥ 12 months) at the time of the study.

Patients with primary SjS are females and a half of them are *de novo* mutations including one case of type II C1INH-HAE. All patients show xerophthalmia (dry eyes) and xerostomia (dry mouth) along with positivity of anti-Ro/SSA and anti-La/SSB antibodies. None of them reports major organ involvement, lymphomas, and/or Central Nervous System complications while two patients have extra-glandular involvement with oligo-articular arthritis and peripheral neuropathy (**Table 2**). In SjS group, LTP occurs in a half of patients (50%, n=2).

Patients with primary APS are mainly women (66.7%, n=2): in all cases, patients show both persistent laboratory evidence of lupus anticoagulant (LA single positive) and recurrent venous thrombosis (along with adverse pregnancy outcomes in one woman); all patients are on vitamin K antagonists (VKA) and 2 patients (75%) are on plasma derived C1INH-LTP at the time of the study.

C1INH-HAE patients with SSc have F:M ratio 1. The SSc-woman has a diffuse cutaneous SSc with clinical vasculopathy (Raynaud’s phenomenon, skin thickening of fingers, digital ulcers), interstitial lung disease, and anti-SCl-70 antibodies: she receives immunosuppressors and iloprost as a long-term treatment to achieve healing and prevention in SSc-related digital ulcers. The SSc-man shows a limited cutaneous SSc with uncomplicated skin involvement (Raynaud’s phenomenon, skin thickening of fingers, telengiectasia), and anti-SCl-70 antibodies. Both of SSc-HAE patients are on LTP at the time of the study.

The single case of MCTD is a woman who has puffy fingers, Raynaud phenomenon, arthralgia, pulmonary arterial hypertension along with anti-U1 ribonucleoprotein (RNP) antibodies. She receives immunosuppressors (including low-dose steroids) and is on LTP at the time of the study.

## Discussion

Our findings provide the first evidence of the prevalence and distribution of RCTDs in a large cohort of C1INH-HAE patients from Italian HAE Centers. The estimated prevalence of RCTDs results to be 2.1%: however, a single RCTD occurs in 0.4-0.5% (SjS, primary APS) and 0.9-1% (SLE) of the study population. Extremely rare diseases such as SSc and MCTD have been registered in 0.2% of cases. The rarity of the aforementioned RCTDs does not allow accurate estimations: results from epidemiological studies are variable due mainly to the heterogeneity of the patients’ populations and/or inclusion criteria (34, 35). Previous evidence reports a prevalence of 12% for autoimmune disease out of 157 individuals with C1INH-HAE but a wide range of diagnoses are included such as glomerulonephritis, inflammatory bowel disease, thyroiditis, inflammatory arthritis, and several CTDs (36). Furthermore, Authors reports six cases out of 143 Italian C1INH-HAE patients having a systemic autoimmune disease (4.2%) but both CTDs and inflammatory arthritis are considered (34). In general, the worldwide SLE prevalence is estimated to be at least 0.04% with the highest prevalence observed in females (37, 38). Specifically, in C1INH-HAE patients, lupus prevalence has been estimated at 2.2% by Donaldson (6, 39). More recently, the French prevalence of lupus among C1INH-HAE patients results between 0.4% and 0.9% of patients: however, authors retrospectively document six cases of type I C1INH-HAE patients with SLE-like or unspecified cutaneous lupus while no cases of defined SLE are described (26). As known, SLE can occur at any age, although it is rare during the first decade of life: however, the risk is particularly high in women of childbearing age (40). Age is thus an independent factor in the onset and diagnosis of SLE: our findings describe a slightly younger mean age at SLE diagnosis with the respect to evidence from the literature (41). Even though adequate comparisons are challenging due to differences in study populations as well as data sources, various hypotheses can explain the difference in mean age at SLE diagnosis. This may be related to factors involved in the pathogenesis of SLE, such as the consumption of complement C4 which occurs in C1INH-HAE with consequent impaired clearance of apoptotic cells (9). Therefore, hypocomplementemia in C1INH-HAE might reduce the age of disease onset of secondary SLE in such patients. In addition to pathogenetic factors, another possible explanation could be the increased likelihood of early detection and diagnosis of SLE in C1INH-HAE patients who are adequately and regularly managed in Tertiary Immunologic settings (41).

According to data from the literature, the overall prevalence of SjS is assumed to be at least 0.4%, including the more common secondary SjS (42). This is in the order of magnitude of our current study (0.46%): however, in our research, we include only the primary form of SjS which is the less prevalent (42). Likewise, the overall estimated prevalence of APS is 0.05% and includes both the primary form and the one associated with other autoimmune diseases, particularly SLE (43, 44). Results from our current study describe a prevalence of 0.35% and refer only to primary APS. In the literature, no data on primary APS in C1INH-HAE have been documented while reports describe APS related to acquired conditions of C1INH deficiency (45, 46).

The global prevalence of SSc has been reported to be 0.023% (47, 48). We describe two cases of SSc-C1INH-HAE who show a different clinical spectrum being classified as limited cutaneous and diffuse cutaneous SSc. Evidence from the literature suggests that CS activation might be involved in the pathophysiology of SSc: abnormal local complement activation has been described in skin lesions of SSc patients, and C3 and/or C4 hypocomplementemia is present in ∼15% of patients with SSc (49, 50). Although it is intriguing to speculate that dysfunctional innate and adaptive immune responses and/or IC might be responsible for CS activation in SSc, evidence from the literature is still debated (51). In our SSc patients, the coexistence of intense C4 consumption related to the congenital C1INH deficiency seems to not correlate with a worse clinical phenotype. However, it must still be considered that these are only two cases for which generalized conclusions cannot be drawn.

MCTD has certainly the lowest prevalence among RCTDs: the reported prevalence estimates are similar across the different criteria sets of MCTD and range from 0.0002% to 0.002% (47, 52). From our findings, the registered MCTD C1INH-HAE patient is a female young adult who shows a defined serological (-U1RNP) and clinical profile also including pulmonary arterial hypertension. As known, women are more likely to develop RCTDs: accordingly, we report a higher F:M ratio among C1INH-HAE with concomitant RCTDs, mainly in SLE and SjS cases. Moreover, the single case of MCTD is a woman.

In C1INH-HAE patients with RCTDs, LTP occurs in a significantly higher prevalence compared with that from HAE patients without RCTDs, suggesting an association between HAE disease activity and systemic autoimmune diseases. However, the rate of patients already in LTP at the time of RCTDs diagnosis is similar to that of patients who start LTP after the RCTD diagnosis. It is plausible to assume that the relation between HAE disease activity and the occurrence of autoimmune diseases in C1INH-HAE might be bidirectional since a higher HAE disease activity can concur to autoimmunity thus increasing the risk of autoimmune diseases by the unregulated consumption of complement C4 (9, 34). Therefore, it is conceivable that more severe forms of HAE, requiring LTP, could be associated with an increased risk of autoimmune diseases. A limitation of the study is represented by the lack of a regression analysis of factors associated to RCTDs that might be useful to describe possible risk factors associated to the development of RCTDs: regression analysis had not been done because of the small sample size that could lead to low accurate estimates and informative results.

In conclusion, we provide the first evidence of distribution of RCTDs in a large cohort of adult Italian patients with C1INH-HAE, documenting that the prevalence of RCTDs - SLE, APS, and SSc - is approximately 5 to 10 times higher in C1INH-HAE patients compared to the general population as reported in the literature. However, in studies on rare diseases, making adequate comparisons is challenging due to potential discrepancies in study populations, data sources, healthcare management, and regional factors. The multicentric design of our study helps to minimize potential bias related to geographic differences, with the aim of providing representative and high-quality data.

According to our results, a greater proportion of patients on LTP occurs among RCTDs patients compared with patients without RCTDs. Elevated HAE activity could promote autoimmunity, increasing the chance of autoimmune conditions by depleting complement C4, which in turn leads to defective clearance of IC. Considering these findings, it’s possible to suggest that the link between HAE and autoimmune diseases might be reciprocal.

## Data Availability

The raw data supporting the conclusions of this article will be made available by the authors, without undue reservation.
